# Parents’ needs during a child’s hospitalisation in a paediatric intensive care unit (PICU): a systematic review

**DOI:** 10.1192/j.eurpsy.2024.1401

**Published:** 2024-08-27

**Authors:** A. Boroklinioti, A. Sarantaki, E. Dousis, A. Zartaloudi, E. Evangelou, C. Dafogianni, M. Polikandrioti, E. Vlachou, I. Koutelekos

**Affiliations:** ^1^ University of West Attica; ^2^Greek Health System, Athens, Greece

## Abstract

**Introduction:**

The admission of children to PICU is a painful experience for parents. Regularly, they are asked to make important decisions about treatment options in collaboration with the care team, which causes them stress, uncertainty and trauma.

**Objectives:**

To investigate the needs of parents during the child’s hospitalization in a pediatric intensive care unit (PICU).

**Methods:**

A systematic review of the literature and a search of articles in the international databases PubMed, Cinahl, Google Scholar, Cochrane Library and Greek scientific journals was performed with a peer review process during the period between April and July 2022. A time limit was set regarding the date of publication of the articles (articles published in the last 15 years).

**Results:**

Nine studies were found that met the criteria for inclusion in the review. The thematic analysis of the results deduced the following sections A: Need for information from health professionals regarding the child’s health status and the possible treatment options available, B: Need for psychological support from health professionals (psychologists, nurses, doctors) in order to be able to manage the difficult situation they are experiencing due to the hospitalization of their child, but also to be able to manage their grief and sorrow in case of loss of the child. C: Need for safe hospitalisation of the child.

**Conclusions:**

Parents have needs during their child’s hospitalization in the PICU, which if put in boundaries-frames and guided by health professionals (who possess knowledge and composure in difficult moments) can bring about a smooth course of the child’s health during hospitalization.

**Disclosure of Interest:**

None Declared

